# Behavioral Interventions to Attenuate Driven Overeating and Weight Regain After Bariatric Surgery

**DOI:** 10.3389/fendo.2022.934680

**Published:** 2022-07-18

**Authors:** Gretchen E. Ames, Afton M. Koball, Matthew M. Clark

**Affiliations:** ^1^ Department of Psychiatry and Psychology, Mayo Clinic, Jacksonville, FL, United States; ^2^ Department of Behavioral Health, Gundersen Health System, La Crosse, WI, United States; ^3^ Department of Psychiatry and Psychology and Division of Endocrinology, Diabetes, Metabolism, and Nutrition, Mayo Clinic, Rochester, MN, United States

**Keywords:** obesity, behavioral treatment, bariatric surgery, reward-based eating, emotional-based eating, impulsivity, weight regain, driven overeating

## Abstract

Weight regain after bariatric surgery is associated with problematic eating behaviors that have either recurred after a period of improvement or are new-onset behaviors. Problematic eating behaviors after bariatric surgery have been conceptualized in different ways in the literature, such as having a food addiction and experiencing a loss of control of eating. The intersection of these constructs appears to be *driven overeating* defined as patients’ experiences of reduced control of their eating which results in overeating behavior. The purpose of this review is to define patient experiences of driven overeating through the behavioral expression of emotion-based eating, reward-based eating, and executive functioning deficits—namely impulsivity—which is associated with weight regain after having bariatric surgery. Delineating concepts in this way and determining treatment strategies accordingly may reduce distress related to the inevitable return of increased hunger, cravings, portion sizes, and tolerance for highly palatable foods after surgery. Along with standard behavioral weight maintenance strategies, topics including acceptance, motivation, emotion-based eating, reward-based/impulsive eating, physical activity, and self-compassion are discussed. These concepts have been adapted for patients experiencing weight regain after having bariatric surgery and may be particularly helpful in attenuating driven overeating and weight regain.

## Introduction

For people who are living with obesity and have a body mass index (BMI) ≥35 kg/m^2^, bariatric surgery is often the most efficacious treatment for achieving and maintaining desired weight loss that is associated with improvements in health and quality of life (1). Unfortunately, providers and patients alike are often uneducated about the efficacy versus the risk of having bariatric surgery, and it remains an underutilized treatment for obesity (2). In examining long-term weight loss, the outcome will vary widely by type of procedure and by patient characteristics (3). A recent systematic review of the literature suggests that patients tend to regain up to 19% of maximum weight loss between 3 and 6 years after Roux-en-Y gastric bypass and potentially more after sleeve gastrectomy (4). Problematic eating behaviors before surgery—although common—are not reliable predictors of suboptimal weight loss and are rarely a contraindication for undergoing bariatric surgery (5, 6). However, weight regain in the long-term is associated with problematic eating behaviors that have either recurred after a period of improvement or are new-onset problems (7–10).

Bariatric surgery is now known to affect regions in the brain associated with motivation, reward, attention, and inhibition in response to food exposure (11). In the first year after surgery, patients may experience reduced cravings, changes in preference for highly palatable foods, and altered responses to environmental food cues (12). Beyond the first year, however, some patients report experiencing increased hunger, food cravings, portion sizes, and tolerance for highly palatable foods (13). Problematic eating behaviors are described in the literature in different, ways including hedonic eating, loss of control eating, food addiction, compulsive eating, binge eating episodes, reward-based eating, emotion-based eating, grazing, and other terms (14). Specifically, food addiction, which is reduced control over food intake with repeated unsuccessful attempts to control intake, and loss of control of eating, defined as the inability to stop or control what or how much food is consumed, have received substantial attention in the bariatric surgery literature (7, 8, 15–17). Still, a lack of consensus remains regarding the uniqueness of these constructs or their clinical utility in terms of diagnosis and treatment before and after having bariatric surgery (18, 19). The intersection of these constructs appears to be *driven overeating* or patients’ experiences of reduced control over their eating, which results in overeating behaviors (20–22). The purpose of this review is to define patient experiences of driven overeating through the behavioral expression of emotion-based eating, reward-based eating, and executive functioning deficits that may be associated with weight regain after having bariatric surgery. Delineating concepts in this way may help guide decision making regarding treatment strategies to attenuate weight regain.

## Emotion-Based Eating

Modern societies have facilitated easy and unending access to highly caloric and palatable foods that can be consumed rapidly. With such easy access, emotion-based eating (i.e., comfort-eating) is prevalent in the general population and likely even more prevalent in those who have obesity (23). Indeed, a recent population-based study demonstrated that emotion-based eating appears to be a plausible pathway between depression and the development of obesity (24). In addition to depression, emotion-based eating is associated with many other factors, including emotion dysregulation, poor emotion regulation skills, low distress tolerance, dietary restraint, loss of control of eating, poor awareness of hunger/fullness signals, body image dissatisfaction, internalized shame, and low self-compassion (24–29). Specifically, a recent study found that patients seeking bariatric surgery who have high levels of internalized shame (e.g., feelings of inferiority, inadequacy, insignificance) and low levels of self-compassion (e.g., disapproving of perceived personal inadequacies, feeling alone in perceived failures) also report high levels of emotion-based eating (25). Thus, increasing self-compassion is likely an important intervention strategy for reducing emotion-based eating. Collectively, research has consistently associated emotion-based eating with longitudinal weight gain, less success with attempts at weight loss, and poorer weight loss maintenance (30–32).

For bariatric surgery patients specifically, emotion-based eating appears to reliably decrease in the first 18 months postoperatively (33, 34). Results beyond the 18-month timeframe are mixed, with some studies suggesting attenuation of emotion-based eating through 36 months after having bariatric surgery and other studies finding reoccurrence between 12 and 36 months after having bariatric surgery (33–39). Emotion-based eating may return postbariatric surgery due to inconsistency of support. While many programs require patients to go through a preoperative nutritional and psychological assessment that includes a focus on problematic eating behaviors, this professional support is often absent postbariatric surgery. Surveys have shown that long-term engagement with bariatric team support diminishes over time, leading to less opportunity for intervention when needed (40, 41). Another reason for a return to emotional eating after having bariatric surgery relates to patients’ dietary patterns and preferences in the first year after having bariatric surgery. While emotion-based eating generally leads people to consume highly palatable foods, patients typically avoid these foods due to care team recommendations and/or due to negative physiological responses to ingestion of high-sugar, high-fat foods (e.g., dumping syndrome) in the months following bariatric surgery. Over time, these physiological responses may diminish, leading to increased poor-quality food consumption (13, 42). A final mechanism in a patient’s return to emotion-based eating after having bariatric surgery may be related to improved emotional states over the short term, leading to less desire or need to use food to cope with negative emotions. Over time, as negative emotional states may return and the “honeymoon period” after having bariatric surgery ends, patients may find themselves craving food to manage negative emotions (43, 44). Taken together, the reoccurrence or new onset of emotion-based eating behavior appears to be an important risk factor for weight regain after having bariatric surgery. At postbariatric surgery follow-up visits, inquiring about emotion-based eating and screening for symptoms of depression and anxiety, as well as other concerning emotions like shame, dissatisfaction with body shape and low self-compassion may help identify patients at high risk for weight regain.

## Reward-Based Eating

Despite societal messages which imply that behaviors necessary to induce weight loss are completely volitional (i.e., eat less and move more), food intake is predominantly determined by one’s neurobiological reward system (i.e., hypothalamus, limbic system, prefrontal cortex) (42). Moreover, gut-brain mechanisms (e.g., leptin and ghrelin regulation) are also strongly related to food intake (45). Thus, many in the obesity scientific community believe that biology, rather than will-power or personal responsibility, is the primary driver of daily decisions about food intake. Two primary components can impact a food’s rewarding influence: (1) “Liking” or experiencing sensory pleasure or enjoyment from food, and (2) “Wanting” or craving foods and being implicitly driven to eat them (46). Also called hedonic eating, or eating beyond what is needed for homeostasis, eating for taste and/or pleasure is associated with weight gain and obesity (47). Neuroimaging studies have found that individuals with higher body weight have enhanced activation of food reward regions in the brain, likely impacting food preference and food choice (45). Some research has also suggested that, compared to normal-weight control groups, those who have obesity have higher levels of reward-based eating (45, 48–51).

After having bariatric surgery, research has shown reduced activation of the brain’s reward systems, indicating that food is inherently less rewarding, and studies have found that reward-based eating is significantly reduced in the first 2 years after having bariatric surgery (52, 53). Postbariatric surgery patients tend to experience less liking of many foods (i.e., tastes and odor preferences changing) and less wanting (i.e., reduce cravings). Moreover, dopamine signaling changes in those who have undergone bariatric surgery, thereby reducing the inherent rewarding properties of many foods (54, 55). After having bariatric surgery, foods that are highly palatable and energy dense are most impacted by these neurobiological changes, which, in turn, decrease food intake (56–59). A recent study demonstrated that for both Roux-en-Y gastric bypass and sleeve gastrectomy, there is a decrease in preference for high fat and calorie-dense foods and an increase in healthy food intake following bariatric surgery (46).

In a qualitative study of weight loss and dietary changes postbariatric surgery, patients described the early months after having bariatric surgery as the “honeymoon period” where “weight loss is easy and surgery does the work” as appetite, hunger, and interest in food are diminished (43). A recent review of the literature on the relationship between bariatric surgery and food preferences highlighted changes in food preferences at varying time points postoperatively (42). In the first year, individuals who had undergone bariatric surgery experienced an increase in their energy intake from proteins, with a decrease in fat consumption through 2 years postoperatively. They also tended to choose more healthy foods and fewer foods containing high-sugar, high-fat, and high-salt content, as well as less calorically dense foods. Despite pleasure ratings for most foods decreasing immediately after bariatric surgery with continued avoidance of high-fat and high-sugar foods beyond the first year postbariatric surgery, food choices seem to eventually (~5 years postop) return to patients’ preferred foods preoperatively (42). More research is needed to confirm the mechanisms by which neurobiological food reward systems regress toward preoperative eating preferences and behavior and the time frame of these potential neurobiological changes. At follow-up visits postbariatric surgery, from a clinical care viewpoint, inquiring about reward-based eating and the return of preference for high-fat and high-sugar foods that can be consumed rapidly may help to identify patients who are at high risk of weight regain.

## Executive Functioning

Impairment in brain systems that activate reward, emotion, impulsivity, motivation for food, decreased inhibitory control and memory functioning have all been implicated as possible pathways in the development of obesity (11). The frontal lobes of the brain are particularly complex as they have extensive and reciprocal connections that integrate information from all other brain regions and are involved in appetite regulation (60). Specifically, the prefrontal cortex is associated with executive functioning, higher-order cognitive processes required to plan and accomplish goals. Deficits in executive functioning have been consistently identified in people living with obesity (61, 62). Broadly defined, executive functioning comprises cognitive flexibility (adapting thinking in response to the environment), working memory (holding small amounts of information in the mind to accomplish complex tasks), verbal fluency (retrieving information from memory), decision making (choosing from multiple possibilities), planning (sequencing thoughts and actions for goal attainment), and inhibition of impulses (delaying gratification).

Impulsivity is a multifactorial personality trait influenced by both genetics and the environment, which is commonly considered to fall under the domain of executive functioning. Two dimensions of impulsivity that are potentially important for understanding eating behavior and obesity are reward-based motivation and behavioral disinhibition (63, 64). For individuals who have obesity and impulsivity, motivation may be connected to eating-related reward pathways in the brain; while behavioral disinhibition is characterized by the occurrence of spontaneous behavior with a little forethought, low effort toward self-regulation, and occurs without regard for consequences. Stated differently, some individuals who have obesity and impulsivity may have high sensitivity to food-related rewards. They may persistently seek out certain foods intended to intensify sensory impact—ultra-processed foods with high-fat, high-sugar, and high-salt content—in anticipation of experiencing reward (63, 65). Moreover, with repeated exposure to highly rewarding foods that are abundant in the environment, the inability to self-regulate consumption results in weight gain.

In addition to food-related reward sensitivity and behavioral inhibition dimensions of impulsivity, negative affect is likely to be another important factor. Specifically, bariatric surgery patients who have impulsive personality traits and symptoms of depression also tend to have high levels of reward-based eating after having bariatric surgery, which is associated with weight regain (66). Schag etal. (66) propose that the pathway between impulsivity and eating behavior is likely mediated by a negative mood. That is, food-related reward sensitivity and behavioral disinhibition of food intake are exacerbated by negative mood states, a construct known as “negative urgency.” Negative urgency, the tendency to act without thought or effort at self-regulation when in distress, is proposed as a third dimension of impulsivity that potentially contributes to weight regain after having bariatric surgery (64, 66). Symptoms of depression typically improve in the first year after having bariatric surgery, but these improvements may erode over time (44), and there is some evidence that many patients increase their antidepressant medications after having bariatric surgery (67). Therefore, patients who have trait impulsivity and experience reoccurrence or new-onset depression symptoms after having bariatric surgery may be particularly vulnerable to reward-based eating when exposed to high-fat and high-sugar content foods.

Bariatric surgery has been proposed as a treatment to improve executive functioning for patients living with obesity (11) (68). Significant improvements in executive functioning occur in the first year and may endure for up to 36 months after bariatric surgery (61, 69). Potential mechanisms for improvement are increased blood flow in the brain, reduced inflammation, alterations in gut hormones, and remission of sleep apnea, hypertension, and diabetes (68). Moreover, there is evidence to suggest that after bariatric surgery, patients who demonstrate the greatest reductions in impulsivity as measured by neuropsychologic testing before and after bariatric surgery also had the greatest reduction in BMI (70). However, with weight regain, increased adiposity, and return of medical comorbidities, there is a risk of reversal of improvements and cognitive decline (69). Research investigating weight regain and executive functioning beyond 4 years after surgery is limited, and clearly more needs to be learned about the long-term benefits of bariatric surgery on executive functioning (71).

Presently, there are no standardized neuropsychological screening tools for measuring executive functioning, including impulsivity postbariatric surgery (61). Screening may be recommended if reasons for nonadherence or poor self-care are unclear or related to deficits in basic comprehension of behaviors required for success postoperatively (72). Other areas of concern may be if patients describe deficits in response inhibition (i.e., impulsive choices regarding highly palatable foods) and deficits in reward processing, such as time discounting (70). That is, the rewards of engagement in weight reduction behaviors (e.g., choosing low-calorie foods) are perceived to be so distant in time that they cease to have any value, thereby exerting little influence on motivation in the present moment. Neuropsychological testing may also be indicated if a diagnosis of Attention Deficit Hyperactivity Disorder (ADHD) is suspected, as there is an association between ADHD and obesity (73).

## Enhanced Behavioral Strategies for Driven Overeating

Maintaining weight loss requires substantial effort as patients experience biological and environmental pressures to regain weight (74). That is, after having achieved nadir weight loss, patients may be unprepared to experience increased hunger, desire for highly palatable foods, increased portion sizes, and driven overeating (13, 75, 76). Standard behavior modification strategies for weight loss have consistently included goal setting, self-monitoring, stimulus control, contingency management, problem solving, thought restructuring, social support, and stress management (77). Self-monitoring remains a core component in obesity treatment and the necessary strategies for weight maintenance are measuring body weight consistently, tracking food intake, and tracking activity level (78, 79).

If patients experience the return of driven overeating after having bariatric surgery, strategies grounded in Acceptance and Commitment Therapy (ACT) may be useful for attenuating weight regain (80). Along with standard behavioral weight-loss strategies, topics discussed below regarding acceptance, motivation, emotional-based eating, reward-based/impulsive eating, physical activity, and self-compassion may also be useful. These concepts have been adapted for patients experiencing weight regain (>10% of nadir weight loss) after having bariatric surgery and may be particularly helpful in reducing driven overeating (81, 82). *Effective Weight Loss Strategies: An Acceptance-Based Behavioral Approach* is an excellent resource for multidisciplinary bariatric surgery care team providers, offering numerous experiential exercises and worksheets for patients (80). Providing a menu of treatment strategies may be most beneficial for supporting patients’ autonomy because not all strategies will be effective for every patient (83). **Table 1** offers a menu of choices for discussion topics and treatment strategies for patients. **Table 2** offers a patient vignette for a 47-year-old woman 18 months post-Roux-en-Y gastric bypass (BMI 27) maintaining a 34% reduction in initial body weight.

**Table 1:** Menu of choices for discussion topics and treatment strategies for patients.

Menu of choices	Discussion topics and treatment strategies
***Acceptance***	Uncontrollable: Reduced appetite and physical restriction lessens over time
	Uncontrollable: Maintenance behaviors become more effortful and less rewarding
	Uncontrollable: Environmental exposure to poor-quality food and sedentary behavior
	Controllable: Self-monitoring behaviors needed for successful weight maintenance
	Controllable: Developing habit strength with frequent, routine, repetitive behaviors
***Motivation***	Remember unpleasant symptoms of living with obesity before surgery
	Connect behaviors with what is most important in life—autonomous motivation
	Reconnect with reasons for initially having bariatric surgery
	Focus on better health, mobility, energy, and quality of life since bariatric surgery
	Identify new challenges that connect with what is most important in life (values)
***Emotion-based Eating***	Eating to avoid negative emotions is ineffective and results in weight gain
	Focus on accepting negative internal experiences (anxiety, wanting certain foods)
	Identify thoughts and expand language around emotional experiences
	Commit to behaviors that connect with values even when rewards are variable
	Practice mindful eating and avoid mindless unplanned eating
***Reward-based/Impulsive Eating***	Contingency management—consider rewards to strengthen desirable behaviors
	Covert sensitization—visualize negative consequences of impulsive eating
	Cue elimination—remove or reduce the visibility of trigger foods from the environment
	Behavioral chain—identify a sequence of events before and after impulsive eating
	Urge surfing—learn to tolerate impulses to eat without acting on them
***Physical Activity***	Identify physical activities that generate positive emotion (joy, pride)
	Identify physical activities that generate social connection
	Focus on how the body feels during physical activity (less joint pain)
	Consider benefits of physical activity beyond weight loss (mood enhancement)
	Focus on increasing light activity and reducing sedentary behavior (sitting, lying down)
***Self-compassion***	Awareness of experiences that are common to the human condition (regret, fear)
	Decrease attachment to negative thoughts and feelings about weight regain
	View loss of focus as a temporary setback rather than a total defeat
	Regain focus on self-monitoring behaviors quickly (the next day when possible)
	Commit to perseverance with behavioral effort

**Table 2:** Patient vignette for 47-year-old female 18 months post-Roux-en-Y gastric bypass (BMI 27) maintaining 34% reduction in initial body weight.

Provider and patient discussion at postsurgery visit	
**Patient**	My cravings for sweets have increased a lot, and I’m worried.
**Provider**	That does sound like something we need to talk about. Tell me about your sweet cravings before you had surgery.
**Patient**	It has always been sugar for me. Whenever I felt stressed or overwhelmed, I would eat candy or really anything sweet. After surgery, I was focused on eating healthy foods because I didn’t want to have dumping or to feel uncomfortable if I ate too much.
**Provider**	The surgery helped you stay on track with your eating.
**Patient**	Yes, I didn’t have sweet cravings and felt in control of my eating. But now, I feel more hunger and sugar cravings are back. It makes me worry that the surgery isn’t working anymore, and I’m losing control of my eating.
**Provider**	Sometimes patients feel unprepared for when the powerful effects of surgery diminish. Often you can eat larger portions, you start to feel hunger again, and you can tolerate a wider variety of foods like sweets. This is common after surgery and doesn’t mean you have messed up or have done something wrong.
**Patient**	Unprepared is right and anxious because I do not want to regain weight.
**Provider**	Describe a recent situation where you had strong sugar cravings.
**Patient**	That’s easy. Last week I had a fight with my husband and on the way home from work I stopped at the grocery store and bought candy that I ate in the car. I haven’t done that in 2 years. I wasn’t even thinking, I just did it.
**Provider**	That must have been distressing for you. You were probably upset both by the negative interaction with your husband and about impulsively eating candy for emotional comfort.
**Patient**	And disappointed because I thought that the surgery would fix my stress eating.
**Provider**	Even after surgery, the reasons people eat are complicated. Some eat for emotional comfort, others prefer the tastes of high sugar and fat foods, or others may make impulsive choices about what to eat. From what we have talked about in other visits, you have achieved everything that you had hoped for with surgery. Your health is great, you are very active, and you are happier with your weight now. It might be good for us to consider alternative coping skills for when you experience negative emotions so that you can feel more in control of your eating. I have some ideas if you are interested?
**Patient**	Ok, yes.
**Provider**	Great, let’s discuss strategies and then you can decide what makes sense to you.

### Promoting Acceptance

Because of the metabolic changes induced by bariatric surgery combined with behavioral effort, patients are typically rewarded with large weight loss and substantial improvements in health within the first year. However, when weight loss diminishes and stops, the powerful effects of reduced appetite and physical restriction induced by bariatric surgery are attenuated (13, 75, 76). Some postbariatric surgery patients may perceive these experiences as unexpected, distressing, and harbingers that their surgical weight loss tool is not working anymore, leaving them vulnerable to weight regain. Furthermore, behaviors that consistently resulted in large weight loss in the first year following bariatric surgery may appear to be more difficult and less rewarding once weight loss diminishes and stops (84, 85). For example, patients may grapple with daily decision fatigue about making healthy food choices or have difficulty with meal planning so that healthy food choices are easily accessible. They may also find it difficult to reduce sedentary behavior. Ultimately the rewards associated with weight loss, such as improved health, mobility, quality of life, and positive attention from others, have lessened and the need for the exertion of long-term focused behavioral effort becomes evident. Therefore, promoting *acceptance* that engaging in weight maintenance behaviors may become more effortful and less rewarding over time may be necessary (80). Similarly, promoting *willingness* means knowing that engaging in healthy eating and physical activity behaviors may be less rewarding than they once were, yet also acknowledging that they are necessary for weight maintenance and continued realization of health and quality-of-life improvements.

Promoting *acceptance* that there are some aspects of weight maintenance that are very challenging or even impossible to control may also be beneficial for some patients (74, 80). For many individuals, fully controlling their environmental exposure to poor-quality foods is unattainable, and the current physical activity environment is such that being sedentary has become the default behavior (86). In the same way, the occurrence of unforeseen life events that may reduce focus on the frequency or repetitiveness of healthy eating and activity behaviors is inevitable (87). For these reasons, one helpful strategy to regain focus may be reconnecting with behaviors that are under personal control, such as self-monitoring. A recent study of nonsurgery patients investigating weight maintenance self-monitoring behaviors compared weight loss maintainers (who lost greater than 24.5 kg and maintained a minimum of 9 kg reduction in weight for more than 3 years) to weight-stable individuals who had obesity (78). Weight loss maintainers commonly endorsed specific behaviors that included keeping a weight graph, setting daily intake goals, having lower-calorie foods easily accessible, choosing lower-calorie food options, measuring portions, and recording food intake. They also appeared to develop habit strength around healthy eating and physical activity. Healthy eating behaviors were done frequently, done as part of a daily routine, or repetitively and done automatically without having to think about them. Inquiring about what postbariatric surgery patients’ current habit strengths are, such as taking vitamins, staying hydrated, focusing on protein intake, or using a sit-to-stand desk at work may help regain focus and build confidence for adding additional weight maintenance behaviors. Regarding physical activity level, weight loss maintainers (BMI 27.6) reduced their sedentary behavior (i.e., sitting or lying down) and spent three more hours per day engaged in light- or moderate-intensity activities (i.e., walking, taking stairs) compared to weight-stable individuals who had obesity (BMI 38.9; total energy expenditure of 1,835 kcal/week vs. 785 kcals per week, respectively) (79). Therefore, reducing sedentary behavior and increasing light activity during leisure time may be a helpful strategy for maintaining weight loss, particularly for postbariatric patients who have low levels of activity and/or dislike engaging in higher intensity physical activities.

### Strengthening Motivational Level for Healthy Living

Discovering *autonomous motivation*—attributing personal values to certain behaviors—may be especially helpful for sustaining behavioral effort to maintain weight loss even when it becomes more difficult and less rewarding (88). One way to do this is to help postbariatric patients reconnect with personal values or their ideas about what is important in life and what experiences have meaning for them. Helping them verbalize the reasons why they decided to have bariatric surgery may initially enhance their current motivational level for healthy living. Weight loss maintainers describe having unpleasant memories about how it was to live with obesity, and these memories can be targeted for high-quality motivation for sustaining behavior change (89). These negative preweight loss memories were primarily concerned with declining physical health as well as shame and embarrassment about body size and shape. Furthermore, weight loss maintainers found achieving improvements in their physical health like the remission of diabetes, lower blood pressure, taking fewer medications, and reduced pain with increased physical activity level as deeply rewarding. They also indicated achieving weight loss inspired increased self-confidence for pursuing personal goals and taking on new challenges (e.g., seeking a promotion at work) that were meaningful and consistent with their personal values. Best practices for conversations about health behavior changes may include a motivational interviewing communication style that includes asking open-ended questions, inviting a wide variety of responses, and offering reflective listening statements that evoke and strengthen the patients’ personal reasons for desiring change (90).

### Reducing Emotional-Based Eating

One aspect of ACT that may be particularly helpful with emotion-based eating is accepting that experiencing thoughts (e.g., dissatisfaction with body shape), emotions (e.g., fear of weight regain, sadness, shame, regret), and physical sensations (e.g., food cravings, hunger) is inevitable (91). The conditioned behavior of eating in response to negative internal or private experiences may appear to be an effective strategy at the moment, but likely increases negative emotion and concern about weight gain in the long term. Eating for emotional comfort is not goal-directed or rational, exposes impulsivity, and ultimately results in undesirable consequences. Rather than trying to change negative thoughts to reduce the intensity of negative emotions (the core concept of cognitive behavioral therapy), patients instead focus on accepting and embracing negative internal experiences that were previously avoided as a normal part of the human experience. Moreover, instead of focusing on problem eating behaviors, patients commit to increasing behaviors that support personal values. In ACT, *committed action* is a concept that refers to maintaining behavior change knowing that the rewards experienced from these behaviors will be highly variable (80). Committed action may be strengthened by exploration of personal values associated with improved health, mobility, and quality of life, like being able to participate in physical activities with loved ones or improving work performance with increased mobility after weight loss.

Another component of ACT includes *mindful eating* (80). Mindfulness, operationally defined in the context of eating, is self-regulation of attention toward physical sensations like cravings, affective states such as anxious mood, and thoughts pertaining to liking and wanting certain foods (92). The second part of mindfulness is having a nonjudgmental attitude of acceptance and curiosity about food-related sensations, feelings, and thoughts. Mindfulness-based approaches have been used effectively for reducing problematic eating behaviors for individuals who have obesity and for bariatric surgery patients (93–95). Specific to weight regain after having bariatric surgery, one study conducted a randomized controlled trial of a 10-session mindfulness-based intervention compared to standard treatment in a sample of postbariatric surgery patients. The treatment was specifically designed to prevent weight gain after having bariatric surgery (95). The mindfulness-based intervention promoted acceptance and reduced reactivity to distress, including learning several different types of meditation (e.g., walking meditation, sitting meditation, or chair yoga) in session and during home practice, group sharing, and didactic sessions. After 6 months, the mindfulness intervention resulted in reduced emotion-based eating but did not result in weight loss. Mindfulness interventions designed to interrupt negative internal experiences and improve decision-making in the moment may help attenuate but not reverse weight gain. Nevertheless, mindfulness training is not yet a part of routine care for bariatric patients, and the long-term benefit and durability of such interventions are not known (96). Acquiring mindfulness skills requires experiential learning and consistent practice, which may not be appropriate or feasible for every patient.

### Reducing Reward-Based/Impulsive Eating

**Figure 1:**
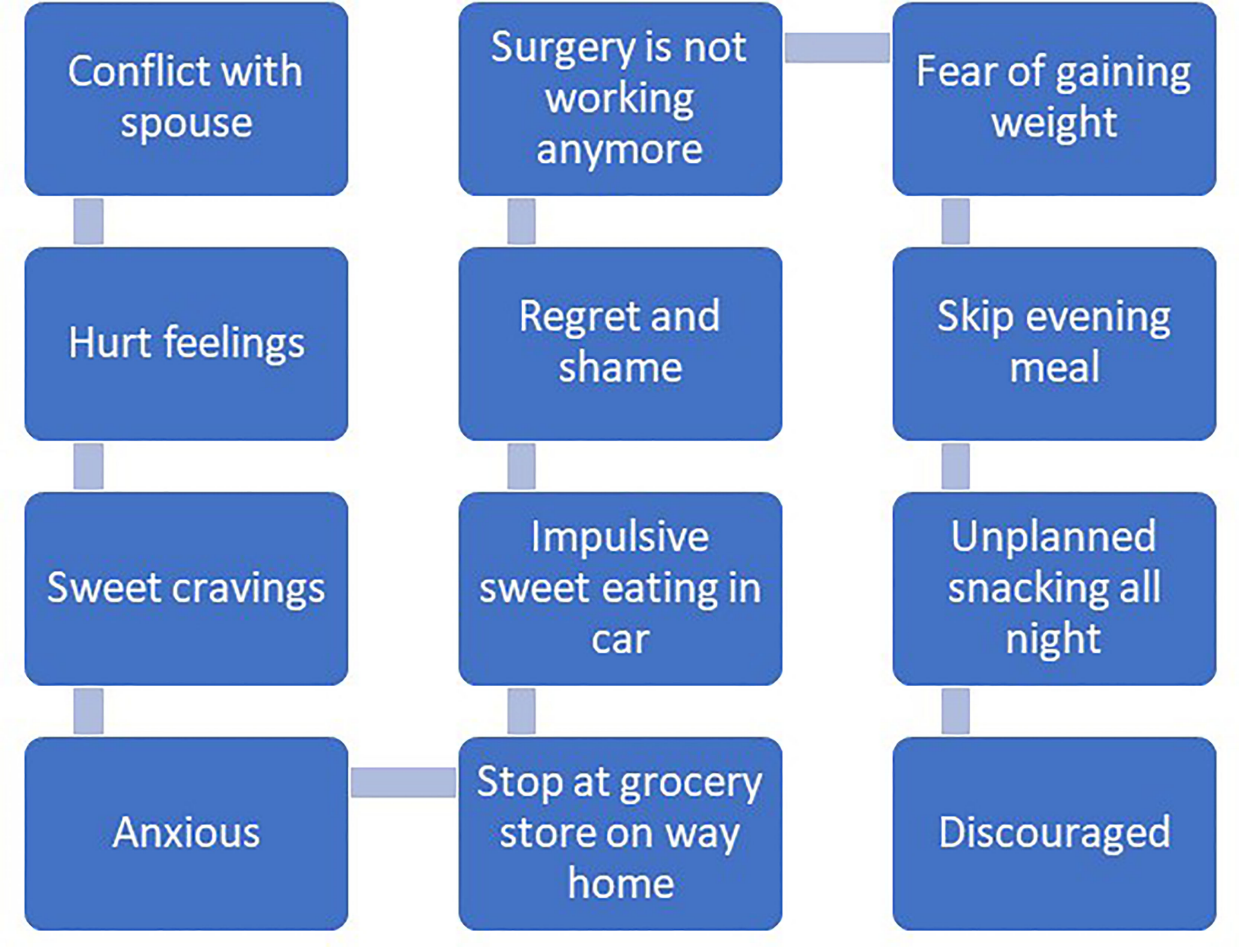
Negative behavioral chain analysis.

**Figure 2:**
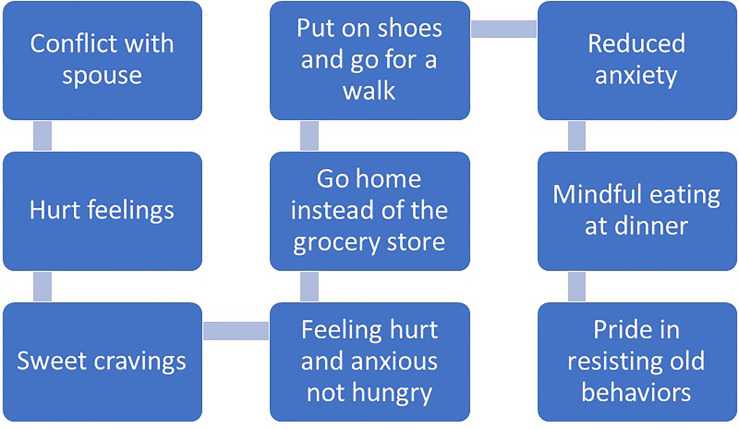
Positive behavioral chain analysis.

Recommendations for managing impulsivity with appetitive behaviors include several specific behavioral strategies (97). First, *contingency management* strategies involve implementing rewards or motivational incentives for desirable behaviors and removing rewards or incentives for undesirable behaviors. Presumably, the anticipated reward of consuming certain foods is to maintain problematic eating behaviors. Patients may consider other rewards that can be introduced or strengthened for performing desirable behaviors unrelated to eating. Second, *covert sensitization* is an imagery exercise designed to associate negative consequences with problem behaviors. Patients may imagine themselves in their usual environment where impulsive eating behavior may occur. Rather than focus on the positive rewards of impulsive eating at the moment (e.g., escape from negative emotion, distraction), patients are instructed to imagine the negative consequences of eating impulsively, like increased emotional distress that is associated with weight regain. If patients successfully reduce the frequency of impulsive eating, emotional distress and weight gain may be lessened over time. Third, *cue elimination* may be particularly effective for some patients as its purpose is to eliminate environmental cues that signal rewards for eating impulsively. Assisting patients with identifying which foods are highly rewarding, triggering impulsive eating behavior and developing management strategies for removal or reduced visibility of those foods may be beneficial. Fourth, assisting patients with *behavioral chain analyses* involves three critical steps for impulsivity management, including (1) identifying a sequence of events that occur immediately before and after impulsive eating, (2) identifying positive consequences that are maintaining impulsive eating as well as the associated negative consequences, and (3) identifying substitute behaviors that are less harmful but notably also perceived as rewarding. **Figures 1**, **2** offer examples of negative and positive behavioral chain analyses for emotion-based and impulsive eating, respectively.

Lastly, it is impossible to avoid foods associated with rewards completely, and experiencing urges, cravings, or impulses to eat is a normal part of the human experience. Therefore, developing management strategies for these inevitable situations is necessary. ACT incorporates concepts of defusion and urges surfing to manage impulsive eating behavior (80). *Defusion* is a concept that suggests viewing urges to eat as a normal part of an internal appetitive experience, and patients have a choice whether to act on the associated thoughts, feelings, and physical sensations. Stated differently, defusion encourages becoming aware of the actual thought process around impulsive eating and observing objectively rather than acting automatically on urges to eat. Similarly, *urge surfing* involves learning to tolerate impulses to eat without acting on them (80, 97). Patients are instructed to imagine urges to eat as ocean waves that build, reach peak intensity, and dissipate over time. Staying in contact with the present moment allows oneself to ride the ocean wave of thoughts, emotions, and physical sensations without judgment until the urge subsides. The goal eventually is that patients may develop what is known as *psychological flexibility.* That is, they are able to tolerate emotions, sensations, and thoughts, good or bad, and behave in accordance with their values of maintaining improved health and quality of life achieved after having bariatric surgery (98).

### Promoting Physical Activity

Recent research suggests that there are themes associated with positive affect (i.e., excitement, determination, pride, sense of accomplishment, joy) while participating in physical activity after having bariatric surgery. These themes included enjoyment of the activity itself, social connection, mindfulness, and mastery of a skill (99). Patients may find it beneficial to reconnect with physical activities that they enjoy earlier in life or perhaps engage in novel activities they have not tried yet, such as yoga or rock climbing. Others may benefit from finding activities that involve connection with others such as a walking group, scheduling a fitness class, or participating in a fitness event like a charity 5-km walk. Mindfulness regarding how physical activity feels in the moment with a healthier body is also associated with positive affect, and many patients will resonate with the term, mindful movement. Patients may reflect on experiencing less joint pain, less shortness of breath, or ease of walking up stairs and focus instead on what their bodies can do after achieving weight loss.

Interestingly, a theme related to negative affect (i.e., frustration, fatigue, dislike) was all-or-nothing thinking about physical activity (99). For instance, focusing only on the negative aspects, having a general dislike of physical activity, or discounting efforts that fall short of personal goals were described as stressful and anxiety-provoking. Reminding patients that while goal setting around reaching a certain frequency, intensity, or time of physical activity is useful, there are other meaningful ways to incorporate activity, like focusing on reducing sedentary behavior during leisure time and increasing light activity throughout the day (79, 99). For patients who have sedentary work lives (e.g., telework), discussing strategies to limit or break up time spent sitting can be beneficial. Another theme that emerged as a barrier to physical activity was using weight loss as the primary motivator to be physically active (99). Considering the known benefits of physical activity beyond weight loss, such as improved metabolic health, fitness, strength, flexibility, balance, and mood enhancement, stress reduction and longevity may be helpful for some patients.

### Promoting Self-Compassion

Destabilizing events that result in loss of control and focus on health behaviors are often unexpected and perhaps not immediately perceived as risk factors for weight gain. These events may include life challenges (e.g., prioritizing caregiving responsibilities over personal health, financial problems, housing insecurity, loss of employment and healthcare benefits), increased appetite, and changes in physical and mental health (e.g., prescribed weight-promoting medication) (13). The experience of weight regain after bariatric surgery is likely to induce feelings of disappointment, frustration, fear of regaining back to their heaviest weight, and shame (13, 75). Shame may be particularly detrimental. It is generated by fear of exposure to increasing adiposity, fear of judgment from others, silence, and secrecy, which may be associated with distancing from possible sources of support, including the bariatric surgery care team. Indeed, patients who have a high degree of internalized shame (e.g., feelings of inadequacy, inferiority) about having obesity may have the lowest levels of self-compassion (25).

*Self-compassion* is defined as increased self-kindness, awareness of experiences common to the human condition, and decreased attachment to negative thoughts and feelings in times of perceived failure, like weight regain (100). A recent investigation of bariatric surgery patients found that a high level of self-compassion presurgery was associated with less depression and greater body image satisfaction, quality of life, and confidence in one’s ability to control eating behaviors 12 months after having bariatric surgery (101). Findings from this study are supported by the larger body of research regarding self-compassion interventions and their impact on a wide variety of psychosocial outcomes. A recent meta-analysis found that there were large effect sizes for self-compassion interventions on improving maladaptive eating behaviors like binge eating and ruminative thinking patterns (102).

Practicing self-compassion in the context of weight maintenance may include acknowledging that loss of control and focusing on health behaviors is an inevitable part of long-term weight management (13, 89). Having days or weeks where there is less focus on the quality and quantity of food consumed or time spent engaging in physical activity should be expected as a normal part of the human experience. How one responds to the loss of focus on weight control behaviors appears to be critically important. Specifically, weight loss maintainers commonly suggested that having “perseverance” with behavioral effort and viewing loss of focus as a temporary setback rather than total defeat was beneficial (89). Moreover, rather than being self-critical, weight loss maintainers regained focus quickly—the next day when possible—by restarting self-monitoring behaviors. Unequivocally, weight loss maintainers accept that tracking food intake is a necessary part of lifestyle change and is critically important for long-term weight loss maintenance.

Finally, helping patients reduce self-judgment by putting into context the status of their current weight may increase self-compassion. Patients may become extremely self-critical about how much weight has been regained from their nadir weight loss. For example, with Roux-en-Y gastric bypass, maintaining a 25% reduction from presurgical weight is excellent, regardless of nadir percent total weight loss, and is often enough to sustain improvements in health status and quality of life (103). Striving to return to the lowest body weight achieved after bariatric surgery may unnecessarily induce emotional distress and self-criticism for some patients. Maintaining a so-called normal body weight or BMI is unnecessary after having bariatric surgery as cardiometabolic health, cancer risk, and life expectancy may improve dramatically even if BMI remains in the obesity range (104–106).

## Conclusion

Identifying eating behaviors that may contribute to weight gain after having bariatric surgery remains challenging. The research literature is consistent that presurgical problematic eating behaviors typically improve in the first 12 months after having bariatric surgery, yet some patients will experience the return of these problematic eating behaviors or new-onset problematic eating behaviors in the long term. The construct of *driven overeating* or reduced control overeating appears to be the commonality among different types of problematic eating behaviors described in the literature. Specifically, patient experiences of driven overeating through a behavioral expression of emotion-based eating, reward-based eating, and executive functioning deficits—namely impulsivity—are associated with weight regain after bariatric surgery. Delineating concepts in this way and determining treatment strategies accordingly may reduce distress related to the inevitable return of increased hunger, cravings, portion sizes, and tolerance for highly palatable foods after surgery. Along with standard behavioral weight-loss strategies, topics such as acceptance, motivation, emotional-based eating, reward-based/impulsive eating, physical activity, and self-compassion may also be useful to the postbariatric surgery patient. These concepts have been adapted for patients experiencing weight regain after having bariatric surgery and may be particularly helpful in managing driven overeating. Providing a menu of treatment strategies may be most beneficial for supporting patients’ autonomy because not all strategies will be effective for every patient.

## Author Contributions

GA and AK conceptualized and wrote the original draft of the manuscript. MC reviewed and edited the manuscript. All authors have read and approved the final draft of the manuscript.

## Conflict of Interest

The authors declare that the research was conducted in the absence of any commercial or financial relationships that could be construed as a potential conflict of interest.

## Publisher’s Note

All claims expressed in this article are solely those of the authors and do not necessarily represent those of their affiliated organizations, or those of the publisher, the editors and the reviewers. Any product that may be evaluated in this article, or claim that may be made by its manufacturer, is not guaranteed or endorsed by the publisher.
